# Elevated HNF1A expression promotes radiation-resistance via driving PI3K/AKT signaling pathway in esophageal squamous cell carcinoma cells

**DOI:** 10.7150/jca.58023

**Published:** 2021-06-16

**Authors:** Naiyi Zou, Xueyuan Zhang, Shuguang Li, Youmei Li, Yan Zhao, Xingxiao Yang, Shuchai Zhu

**Affiliations:** 1 Department of Radiation Oncology, The Fourth Hospital of Hebei Medical University, Shijiazhuang, Hebei 050011, P.R. China.; 2 Department of Infection Management, The Fourth Hospital of Hebei Medical University, Shijiazhuang, Hebei 050011, P.R. China.

**Keywords:** ESCC, HNF1A, proliferation, metastasis, γH2AX, PI3K/AKT pathway

## Abstract

**Purpose:** Radiotherapy is a major modality for treatment of local advanced esophageal squamous cell carcinoma (ESCC). Hepatocyte nuclear factor 1-alpha (HNF1A) is involved in regulation of tumor cell proliferation, apoptosis, cycle distribution, invasion metastasis and chemical resistance. The aim of this study was to investigate the effect of HNF1A on radiosensitivity of ESCC cells.

**Methods:** In our study, HNF1A expression was verified from GEPIA in multiple types of cancer. The prognostic value of HNF1A in ESCC was obtained by TCGA database. In addition, the expression of HNF1A in ESCC cell lines was verified by western blot. Subsequently, lentiviruses were used to construct HNF1A overexpressed cell lines TE1 and KYSE150.Then, the roles of HNF1A on cell proliferation, invasion, apoptosis, cell cycle distribution and radiosensitivity were verified. Furthermore, the relationship between HNF1A and γH2AX were determined by western blot and immunofluorescence. We also detected the expression changes of key factors in PI3K/AKT pathway after overexpression of HNF1A.

**Results:** The results showed that the overexpression of HNF1A promoted cell proliferation and invasion with or without irradiation (IR), and potently radiation-resistance ESCC cells with a sensitization enhancement ratio (SER) of 0.76 and 0.87. In addition, HNF1A regulated Cyclin D1 and CDK4 proteins to promote the transition from radiation-induced G0/G1 phase arrest to S phase, and coordinated BAX and BCL2 proteins to reduce the occurrence of radiation-induced apoptosis. It was worth noting that HNF1A might be involved in radiation-induced DNA damage repair by regulating γH2AX though PI3K/AKT signal pathway.

**Conclusion:** Our study preliminarily suggested that HNF1A was associated with the progression and radiosensitivity of ESCC cells, and it might reduce the radiosensitivity of ESCC cells by promoting cell proliferation, releasing G0/G1 phase arrest, reducing apoptosis, and regulating the expression of γH2AX protein though driving PI3K/AKT signal pathway.

## Introduction

Esophageal cancer is a malignancy neoplasm of digestive tract and the sixth leading cause of death from cancer and the seventh most common cancer globally, mainly because of its extremely aggressive nature and poor survival rate 1. The incidence and mortality of esophageal cancer are also influenced by geographic location. ESCC principally occurs in the “Asian Esophageal Cancer Belt” 2. In a 2015 Chinese cancer epidemiology report, the death rate of esophageal cancer ranked fourth 3. In recent years, radiotherapy has made tremendous progress in the treatment of esophageal cancer in all aspects of clinical practice 4. However, the main reason for the failure of chemoradiotherapy is recurrence. The most common sites of recurrence are locoregional areas (44.5%), distant lymph nodes (19%), and distant organs (15.9%) 5. The precise molecular mechanism underlying enhance the radiosensitivity of ESCC are only partially understood, resulting in limited benefit to patients with ESCC from radiotherapy. Therefore, improving the sensitivity of ESCC to radiation has become the focus of oncologists.

HNF1A belongs to the HNF1 family, was located on human chromosome 12q24.3, and is a transcription factor containing a homologous domain 6. There is a controversy for the biological role of HNF1A in pancreatic cancer (PC). The researchers identified HNF1A as a central transcriptional regulator of both pancreatic cancer stem cells (PCSC) properties and a new oncogene in pancreatic ductal adenocarcinoma (PDA) 7. However, HNF1A is also reported to down-regulate the cell cycle and anti-apoptotic genes, and up-regulate the expression of apoptotic genes 8, inhibits to induce the resistance of pancreatic cancer cells to gemcitabine by targeting ABCB1 9, and regulates the proliferation activity of pancreatic cancer cells by increasing the PTEN/AKT signaling pathway 10. These findings strongly support the role of HNF1A as a tumor suppressor in PC. In other studies, researchers have found that the combination of HNF1A, HNF4A and fork head box protein A3 (FOXA3) can reprogram hepatocellular carcinoma cells into hepatocyte-like cells 11. In addition, another study showed that the combined expression of these three transcription factors inhibited the growth of hepatocellular carcinoma cells 12. At the same time, silencing miR-484 can be down HNF1A/MMP14 and inhibited the growth and metastasis of tumor cells, on the other hand, the overexpression of HNF1A/MMP14 promoted the adhesion and EMT process in cervical cancer 13. Researchers found that HNF4G/HNF1A was highly expressed in prostate cancer, and that the lack of HNF4G/HNF1A inhibited the growth of prostate cancer cells 14. These studies suggest that HNF1A is involved in the proliferation and development of multiple tumors, but performs different functions. However, few researchers have studied the biological function of HNF1A in ESCC, and the association with radiosensitivity has not been reported.

The aim of this study was investigate the effects of HNF1A on biological function and radiosensitivity of ESCC cells. Based on our incipient test results, we designed following experiments to explore the effect mechanism of HNF1A on the radiosensitivity of ESCC cells *in vitro*, and further translated the affect results for clinical outcome.

## Material and methods

### Cell culture and X-ray irradiation

Human esophageal epithelial cells (HEEC) and human ESCC lines (ECA109, TE13, KYSE30, TE1 and KYSE150) were cultured in RPMI-1640 medium supplemented with 10% fetal bovine serum (FBS) and 1% penicillin/streptomycin at 37 °C in 5% CO2. All cells were proved form Scientific Research Center of the fourth Hospital of Hebei Medical University.

The ESCC cells were irradiated by a 6-MV Siemens linear accelerator (Siemens, Buffalo Grove, IL, USA) at room temperature. The source-skin distance (SSD) was 100 cm and the dose rate was 500 MU/min. The cells continued to be cultured until they were collected a specific time for further study.

### Cell transfection

The HNF1A overexpression lentivirus was purchased from GeneChem Co., Ltd (Shanghai, China). The cells (5×10^5^/well) were cultured in 6-well plates until they reached 50% confluency, and then the overexpression lentivirus and the negative control (NC) lentivirus were added to them for 24 h according to instructions. The transfection efficiency was observed by fluorescence microscopy after 72 h. Puromycin of 2 μg/ml was used to screen the cells and then subcultured.

### Real-time quantitative polymerase chain reaction (RT-qPCR)

Total RNA was extracted from esophageal tumor cells using TRIzol reagent (Thermo Fisher Scientific, Inc.). Then the total RNA was reversed-transcribed to cDNA with a RevertAid First Strand cDNA Synthesis Kit (Thermo Fisher Scientific, Inc. cat. K1622) at 42 °C for 60 min and 70 °C for 5 min. RT-qPCR was then performed using MonAmp™ SYBR^®^ Green qPCR Mix (Monad Biotech Co., Ltd.) to analyze the expression of the HNF1A gene according to the manufacturer's protocol. Subsequently, the samples were initially denatured at 94 °C for 30 sec, followed by 40 cycles of the repeated procedure as follows: denaturation at 94°C for 5 sec, annealing at 56 °C for 15 sec and extension at 72 °C for 10 sec. As a control, the levels of glycerol-dehyde phosphate dehydrogenase (GAPDH) expression were also analyzed. The 2^-ΔΔCT^ method was applied to analyze HNF1A gene expression 15. The following primers were designed and used for RT-qPCR:

- GAPDH forward: 5′-CGCTGAGTACGTCGTGGAGTC-3′;
- GAPDH reverse: 5′-GCTGATGATCTTGAGGCTGTTGTC-3′;
- HNF1A forward: 5′-GAGGACGAGACGGACGACGAT-3′;
- HNF1A reverse: 5′-TGCCCTTGTTGAGGTGTTGG-3′.

### Western blot analysis

Protein expression was also evaluated using western blotting in protein samples extracted from cell lines. Western blot analyses were performed as previously described 16. The relevant antibodies were anti-HNF1A (dilution 1:500; cat. no. sc-135939; Santa cruz biotechnology, Inc.), anti-γH2AX (dilution 1:1,000; cat. no. ab81299; Abcam), anti-MMP2 (dilution 1:2,000; cat. no. ab97779; Abcam), anti-MMP9 (dilution 1:5,000; cat. no. ab76003; Abcam), anti-cyclin D1 (dilution 1:200; cat. no. ab16663; Abcam), anti-CDK4 (dilution 1:2,000; cat. no. ab199728; Abcam), anti-BAX (dilution 1:5,000; cat. no. 60267-1-Ig; Proteintech), anti-BCL2 (dilution 1:2,000; cat. no. 60178-1-Ig; Proteintech), anti-β-actin (dilution 1:5,000; cat. no. 60008-1-Ig; Proteintech), anti-E-cadherin (dilution 1:2,000; cat. no. 60335-1-Ig; Proteintech), anti-Vimentin (dilution 1:4,000; cat. no. 10366-1-AP; Proteintech), anti-N-cadherin (dilution 1:2,000; cat. no. 22018-1-AP; Proteintech), anti-p-AKT (Ser473, dilution 1:1,000; cat. no. 4058; Cell Signaling Technology, Inc.), anti-AKT (dilution 1:1,000; cat. no. 4685; Cell Signaling Technology, Inc.), anti-PI3K p110β (dilution 1:1,000; cat. no. ab151549; Abcam). Then, the blotted protein bands were observed by the Odyssey system (LI-COR Biosciences, Lincoln, NE, USA). To observe the changes in the protein expression level, we calculated the ratio of the protein to the corresponding β-actin. Three separate experiments were conducted for each western blot analysis.

### Cell proliferation assay

After cell counting, the cells were diluted to 5×10^4^ cells/ml and 100 µl cell suspensions were seeded into each well in 96-well plates with 5 duplicates for each sample. When the cells adhered, they were irradiated. Then 10 µl/well Counting Kit-8 (Med Chem Express (MCE) Princeton, NJ, USA) was added at 24 h, 48 h and 72 h, and the absorbance was detected after 1 h at 450 nm via Multiskan. The experiments were repeated at least three times before analysis.

### Colony formation assay

The ESCC cells were irradiated at doses of 0, 2, 4, 6 and 8 Gy and then plated in 6-well plates with appropriate cell numbers. After 14 days, the cells were fixed with paraformaldehyde (4%) and stained with crystal violet (0.1%). The cell colony (> 50 cells) count was then performed. The results were analyzed through GraphPad Prism version 5.0 (GraphPad Software, Inc., La Jolla, CA, USA), and the biological parameters of radiation and survival curves were obtained.

### Transwell assay

The invasive ability of ESCC cells was detected by transwell assay. RPMI-1640 medium (200 µl) without FBS containing 1×10^4^ cells were added to the transwells precoated with Matrigel (both from Corning Inc., Corning, NY, USA), and 800 µl RPMI-1640 medium with 10% FBS was added to the lower chamber. After 24 h, cotton swabs were used to wipe the cells on the upper side of the chamber and the cells on the lower side of the chamber were fixed with paraformaldehyde (4%) and stained with crystal violet (0.1%) for 10 minutes. Then, they were photographed with a fluorescence microscope (Nikon Ti2, Japan). The stained cells penetrating the chamber were counted and used to analysis.

### Analysis of cell cycle distribution and apoptosis

To observe the effect of HNF1A on the cell cycle and apoptosis, flow cytometry (FCM) was used. The transfected cells were irradiated, and the cells were collected according to the instructions after 48 h. The cells were stained with propidium iodide solution (MultiSciences Biotech Co., Ltd.) to detect cell cycle distribution. Annexin V and 7-AAD (BD Biosciences, San Jose, CA, USA) were used to measure the apoptosis rate.

### Immunofluorescence Staining

We used PBS to rinse the cells on glass coverslips 3 times for 3 minutes. Next, the cells were fixed with 4% paraformaldehyde for 10 minutes. We used PBS to wash it 3 times. Then, the cells were treated with 0.5% Triton X-100 for 15 minutes. To block nonspecific binding, we incubated cells with 10% goat serum for 30 minutes. At 4 °C, the cells were incubated overnight with primary antibodies. According to observation conditions fluorescent secondary antibodies (dilution 1:100; cat. no. SA00013-2 and SA00013-3; Proteintech) were selected on the second day. Since the virus-transfected cell lines produced green fluorescent protein (GFP), we observed the expression of γH2AX using a red fluorescent secondary antibody (dilution 1:100; cat. no. SA00013-4; Proteintech) after transfection. To observe protein localization, we used DAPI (cat: no. C0065; Beijing Solarbio Science & Technology Co., Ltd.; China) for nuclear staining. Finally, we use a laser confocal microscope (Nikon A1, Japan) to take pictures.

### Gene Expression Profiling Interactive Analysis

GEPIA (Gene Expression Profiling Interactive Analysis), a Web-based tool to deliver fast and customizable functionalities based on TCGA and GTEx data 16. According to the operation instructions of GEPIA database, we carried out gene expression analysis, gene correlation analysis, patient survival analysis and correlation analysis between gene and clinical stage. GEPIA was available at http://gepia.cancer-pku.cn/.

### Kaplan-Meier plotter

The background database was manually curated. Gene expression data and relapse free and overall survival information were downloaded from GEO, EGA and TCGA. The two patient cohorts were compared by a Kaplan-Meier survival plot, and the hazard ratio with 95% confidence intervals and log-rank P value are calculated 17. The Kaplan-Meier plotter was available at https://kmplot.com/analysis/.

## Results

### HNF1A was associated with poor prognosis of ESCC

**Figure 1:**
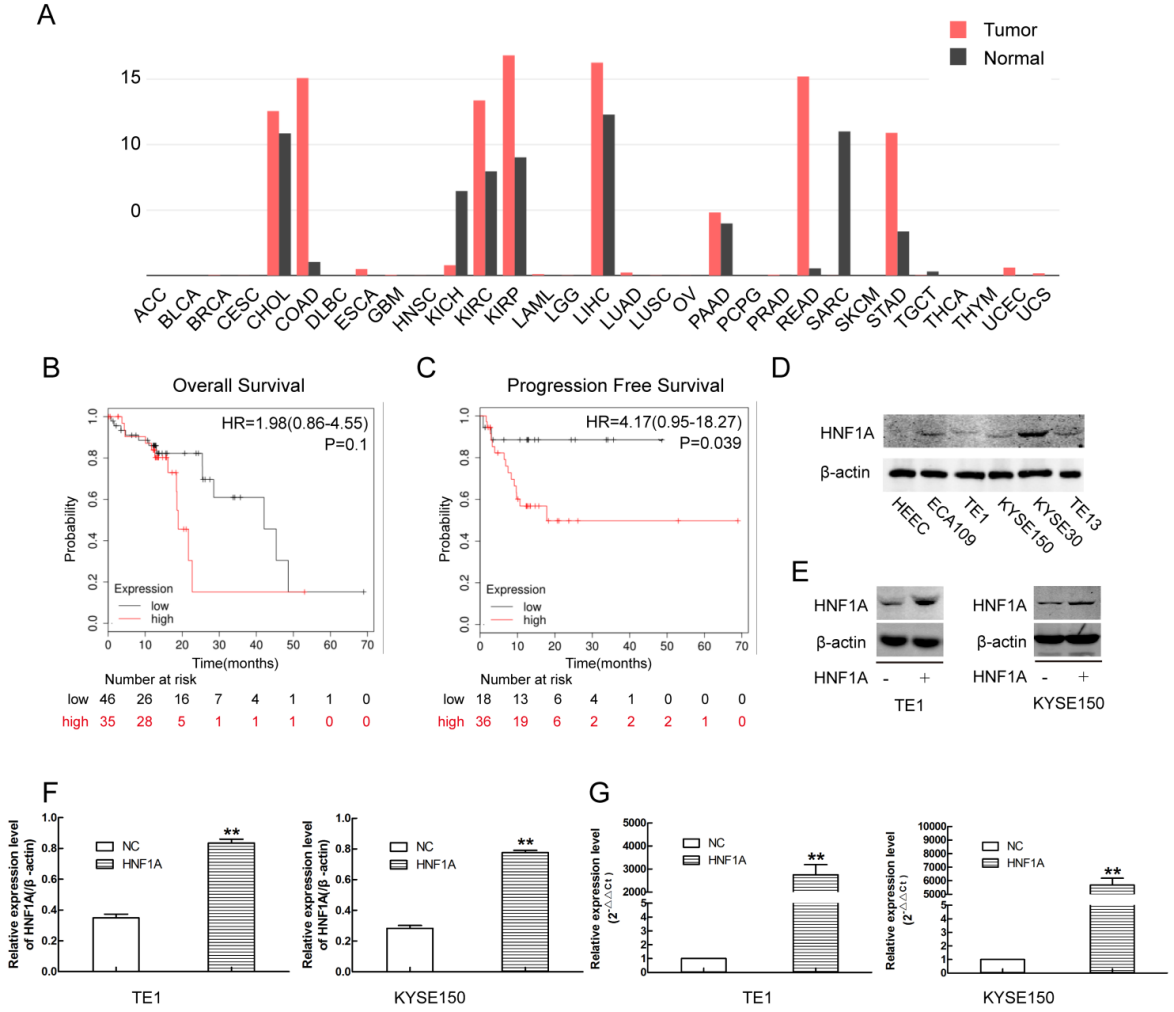
There was a relation between HNF1A and prognosis of the patients with ESCC, and the construction of HNF1A overexpression cells line by using overexpression lentivirus. **(A)** HNF1A expresses in various tumors. **(B, C)** HNF1A was related to OS and PFS of patients with ESCC. **(D)** The expression of HNF1A in ESCC cell lines and HEEC cells. **(E, F)** HNF1A overexpressed cell lines TE1 and KYSE150 were successfully constructed at the protein level. **(G)** At the mRNA level, HNF1A mRNA expression in the HNF1A group was obviously higher than that in the NC group. **P < 0.01; HNF1A: hepatocyte nuclear factor 1-alpha; ESCC: esophageal squamous cell carcinoma; HEEC: human esophageal epithelial cells; OS: overall survival; PFS: progression-free survival.

Through online analysis of GEPIA database, we obtained the expression of HNF1A in a variety of tumors (Fig. 1A). The expression of HNF1A was high in ESCC compared with normal tissues. Kaplan-Meier plotter database showed that the patients of ESCC with high expression of HNF1A have a poor OS (N=81, P=0.1) and PFS (N=54, P=0.039) (Fig. 1B, C). The expression of HNF1A was higher in ESCC cells than in human esophageal epithelial cells (HEEC) (Fig. 1D). HNF1A was highly expressed in most tumor tissues and associated with prognosis of cancer patients. So we had a strong interest in the biological function of HNF1A.

### Ectopic expression of HNF1A promoted proliferation and radiation-resistance of ESCC cells *in vitro*

**Figure 2:**
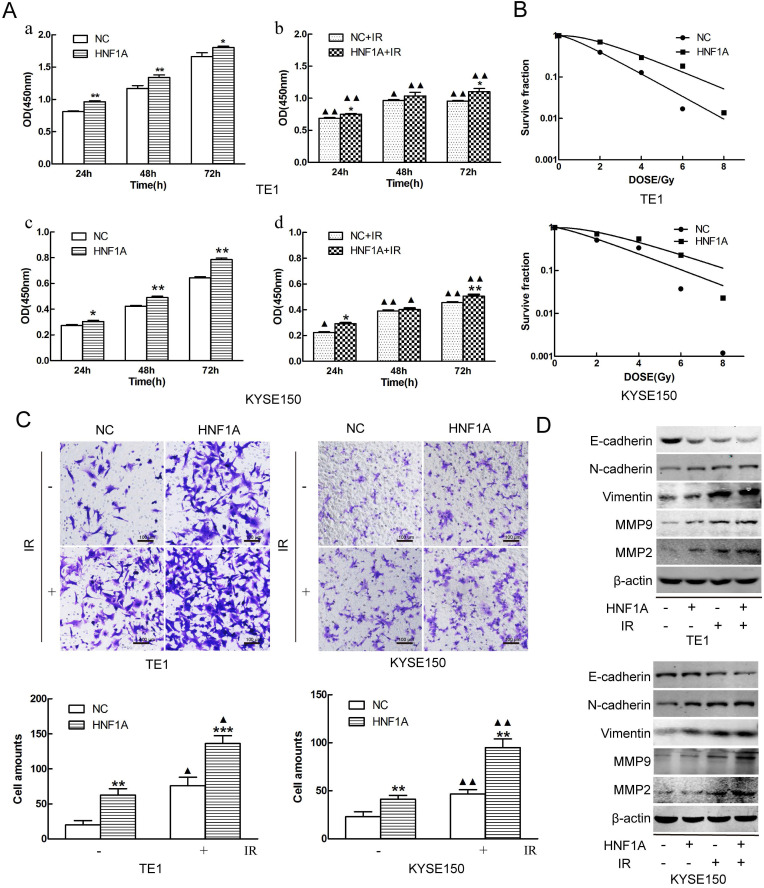
HNF1A increased the proliferation and invasion of TE1 and KYSE150 ESCC cells, and reduced their radiosensitivity. **(A)** Cell proliferation was detected by the CCK8 assay. **(B)** The radiosensitivity of ESCC cells was measured by clone formation assay. **(C)** Transwell assay was used to detect the ability of cell invasion (magnification, x200). **(D)** The EMT-related proteins and MMP2/MMP9 proteins were detected by western blot. A comparison with the NC group is indicated by asterisks *P < 0.05, **P < 0.01 and ***P < 0.001; a comparison with the corresponding non-irradiated group is indicated by triangles ▲ P < 0.05 and ▲▲P < 0.01. CCK-8: Cell Counting Kit-8; HNF1A: hepatocyte nuclear factor 1-alpha; IR: irradiation; ESCC: esophageal squamous cell carcinoma; EMT: epithelial mesenchymal transition.

In order to investigate the mechanism of HNF1A regulated the prognosis of ESCC. We selected TE1 and KYSE150 cells with relatively low HNF1A expression for subsequent experiments. We used HNF1A overexpression lentivirus to infect TE1 and KYSE150 cells, and constructed with HNF1A overexpression cell lines. They were verified at the RNA and protein levels which increase at least double (Fig. 1E, F and G). To observe the effect of HNF1A on the viability, the CCK-8 assay was used to detect cell proliferation at 24, 48 and 72 h after IR (Fig. 2A). The results showed that the viability of HNF1A groups were obviously higher than that of NC groups at any time with and without IR (P < 0.05) (Fig. 2Aa, b, c and d). After IR, all groups cells proliferation was significantly decreased (P < 0.05). In addition, we employed the colony formation to detect radiosensitivity of ESCC cells (Fig. 2B). Compared with NC group, the survival curves of the TE1 and KYSE150 HNF1A overexpression groups were significantly shift, with a SER 0.76 and 0.87, respectively. Finally, our findings indicated that HNF1A Ectopic expression significantly promoted proliferation and radiation-resistance of ESCC cells.

### HNF1A mediated epithelial mesenchymal transition (EMT) to regulate the ability of cell invasion

Metastasis was a significant process in the development of malignant tumors, and it was necessary to detect the invasion ability of tumor cells. The invasion of the ESCC cells was observed by transwell assay. The results revealed that overexpression of HNF1A enhanced the cell invasion of TE1 and KYSE150 cells with or without IR (Fig. 2C). Compared with the NC group, the number of cell invasion in the HNF1A group increased (P < 0.05) with or without IR. In addition, we observed the expression of EMT-related proteins and MMP2/MMP9 by western blot. These results showed that the expression of E-cadherin was decreased in HNF1A group with or without IR, but the expression of Vimentin/N-cadherin/MMMP2/MMP9 were the opposite (Fig. 2D). EMT not only regulated the invasion of tumor cells, but also were involved in double-strand DNA repair, the escape from senescence and the induction of an anti-apoptotic and pro-survival phenotype 18. These revealed that HNF1A may be associated with EMT to influence cells invasion or even radiation-induced DNA double-strand breaks (DSBs) repair.

### Overexpression of HNF1A decreased the apoptosis rate of ESCC cells after IR

**Figure 3:**
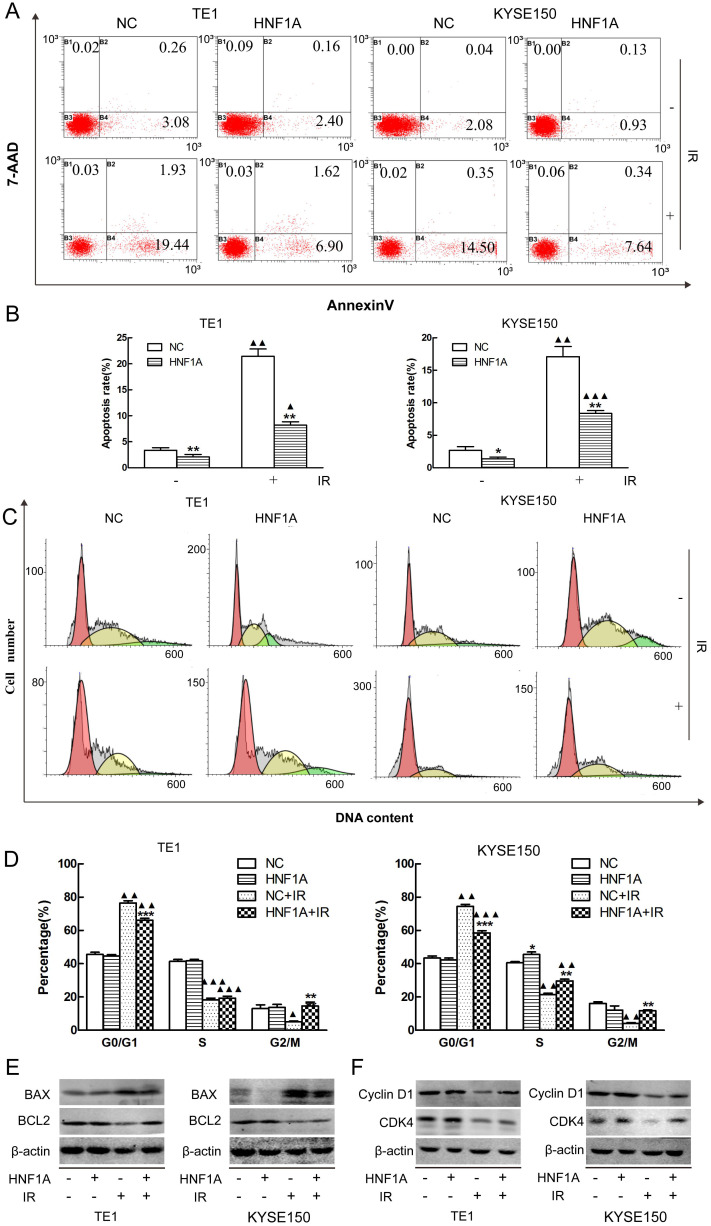
HNF1A regulated the apoptosis rate and cell cycle distribution of TE1 and KYSE150 ESCC cells. **(A, B)** The apoptosis rate in each group was detected by flow cytometry. **(C)** HNF1A relieved G0/G1 phase arrest in TE1 and KYSE150 ESCC cells after IR. **(D)** The histogram of cell cycle distribution was expressed as mean ± standard error for each group. **(E)** After IR, the proteins BAX/BCL2 were regulated by HNF1A in TE1 and KYSE150 cells. **(F)** HNF1A regulated cyclin D1/CDK4 to relieve G0/G1 phase arrest after IR. A comparison with the NC group is indicated by asterisks *P < 0.05, **P < 0.01 and ***P < 0.001; a comparison with the corresponding non-irradiated group is indicated by triangles ▲ P < 0.05, ▲▲P < 0.01 and ▲▲▲P < 0.001. HNF1A: hepatocyte nuclear factor 1-alpha; IR: irradiation.

Radiation-induced apoptosis was a mechanism by which radiation kills tumor cells. We used FCM to observe the changes of radiation-induced apoptosis. Our results showed that the apoptosis rates were significantly difference between HNF1A group and NC group with or without IR (Fig. 3A, B). The apoptosis rate was less in the HNF1A group of TE1 (P = 0.008) and KYSE150 (P = 0.043) without IR. After IR, the apoptosis rate increased in TE1 NC group (P = 0.002), HNF1A group (P = 0.009) and KYSE150 NC group (P = 0.003), HNF1A group (P = 0.000). However, compared with NC group, HNF1A group had a lower apoptosis rate of TE1 (P = 0.006) and KYSE150 (P = 0.006) cells after IR. To further prove the ectopic expression of HNF1A could reduce the irradiated-induced apoptosis. We turned our attention to apoptosis-related proteins, including BAX and BCL2. Results showed that BAX was increased and BCL2 was decreased after IR. However, when HNF1A was overexpressed, BAX decreased and BCL2 increased with or without IR (Fig. 3E). Our investigation demonstrated overexpression of HNF1A down-regulated the apoptosis of radiation-induced.

### HNF1A relieved G0/G1 phase arrest caused by IR in ESCC cells

Cell cycle detection was essential to understand the mechanism by which HNF1A affected the radiosensitivity of ESCC cells. We detected cell cycle distribution by FCM. Our findings showed that TE1 and KYSE150 cell cycle were arrested at the G0/G1 stage in both NC group (P = 0.002, P = 0.002) and HNF1A group (P = 0.001, P = 0.000). Although G0/G1 stage arrest was observed in all groups after IR, the G0/G1 arrest was significantly decreased in HNF1A group compared with NC group in TE1 (P = 0.000) and KYSE150 (P = 0.000) cells. Interestingly, after IR, the G2/M phase ratio of NC group were decreased in TE1 (P = 0.049) and KYSE150 (P = 0.004) cells. But the G2M phase ratio of HNF1A group were not significantly decreased in TE1 (P = 0.421) and KYSE150 (P = 0.841) cells (Fig. 3C, D). We future analyzed a panel of cell cycle regulatory proteins by western blot analysis following ectopic expression of HNF1A. The results showed that expression of Cyclin D1 and CDK4 were increased in HNF1A overexpression group compared with NC group with or without IR. Notably, expression of Cyclin D1 and CDK4 were decreased after IR (Fig. 3F). As a consequence of DNA damage, Cyclin D1 was degraded and Cyclin D-CDK4/6 complexes were destroyed, thereby causing to cell cycle arrest 19. We found that HNF1A could reverse the radiation-induced degradation of Cyclin D1, that contributing to remove the G0/G1 block and promote cell cycle progression. From these results we hypothesized that HNF1A was involved in the DNA damage repair process, for this reason resulting in radiation resistance. Combined with the above results, HNF1A appeared to be associated with DNA damage repair. Therefore, we hypothesized that HNF1A affected the radiosensitivity of ESCC cells by participating in DNA damage repair.

### HNF1A played an important role in the DNA damage repair after IR by initiating the PI3K/AKT signaling pathway

**Figure 4:**
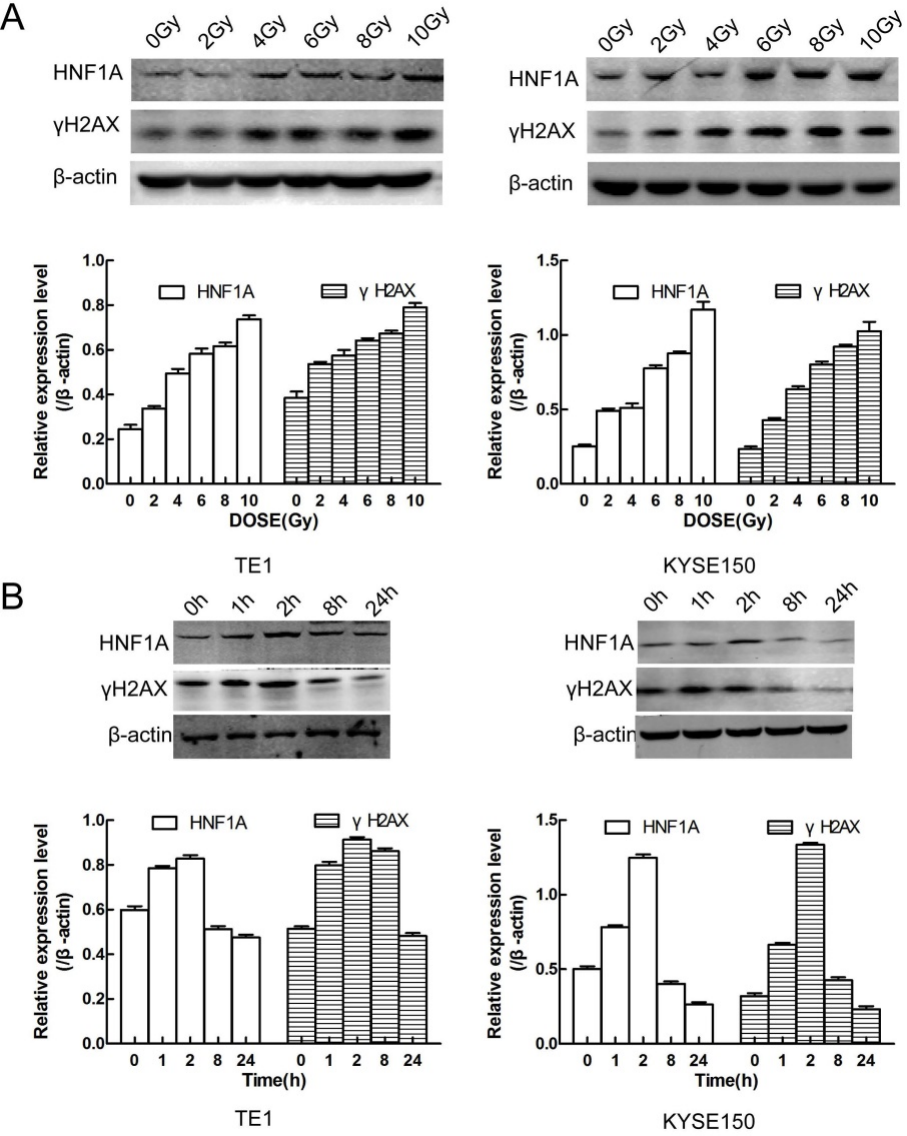
It showed that relationship between HNF1A and γH2AX protein expression and radiation exposure time and dose. **(A)** The protein expression of HNF1A and γH2AX varied with the radiation dose in cells. **(B)** There was a relation with time between HNF1A andγH2AX proteins after 6Gy IR. IR: irradiation; HNF1A: hepatocyte nuclear factor 1-alpha.

**Figure 5:**
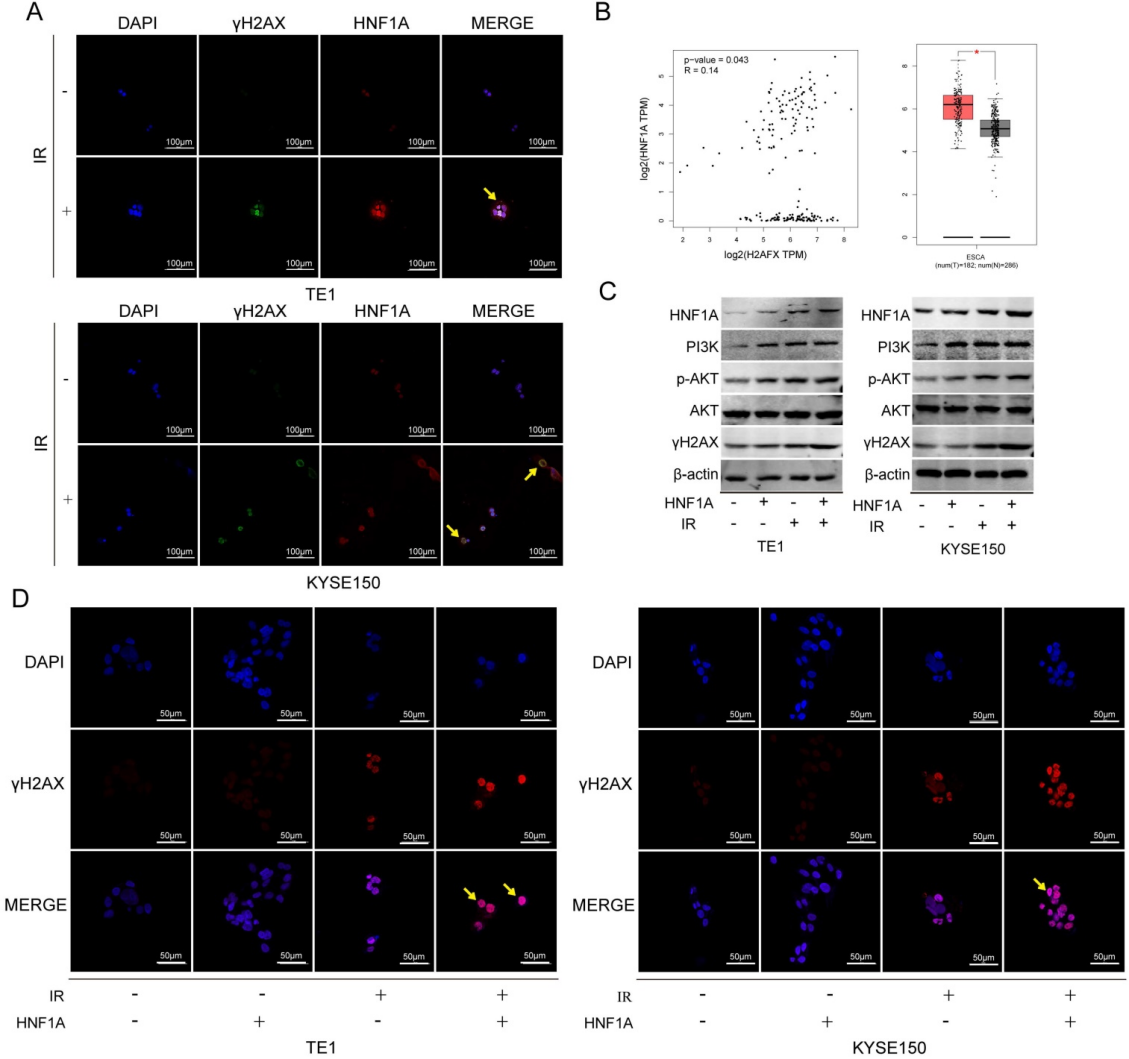
HNF1A participated in DNA damage repair by regulating the expression of γH2AX protein, and played a carcinogenic role by activating PI3K/AKT signaling pathway. **(A)** Subcellular localization of HNF1A and γH2AX proteins (magnification, x400). **(B)** Through GEPIA online analysis, the relationship between HNF1A and γH2AX was obtained, and the expression of γH2AX in tumor tissues was higher than that in normal tissues. **(C)** The expression of HNF1A/PI3K p110β/p-AKT/AKT/γH2AX proteins were detected by western blot. **(D)** After ectopic expression of HNF1A, the number of γH2AX focus formations was observed by immunofluorescence assay (magnification, x600). HNF1A: hepatocyte nuclear factor 1-alpha; GEPIA: gene expression profiling interactive analysis.

We first thought of the γH2AX protein, which was a key signal for identifying DNA DSB and initiating DNA damage repair 20. In TE1 and KYSE150 cells, the proteins of HNF1A and γH2AX were positively correlated with IR dose (Fig. 4A), and they reached the highest level after 2 h IR, and then gradually decreased (Fig. 4B). HNF1A and γH2AX showed consistent changes after IR, so we hypothesized that HNF1A was involved in regulating radiation-induced γH2AX protein expression to accelerate DNA damaged repair, thereby reducing radiosensitivity of ESCC cells. To test this hypothesis, we observed the changes of γH2AX after ectopic expression of HNF1A with or without IR. Immunofluorescence was used to observe the expression and localization of HNF1A and γH2AX in TE1 and KYSE150 cells (Fig. 5A). The results showed that HNF1A expressed in the cell nucleus and cytoplasm, but a stronger red fluorescence was observed in the cytoplasm after 2 h IR, indicating that radiation could induce the release of HNF1A from the cell nucleus into the cytoplasm. The number of γH2AX focus formation was increased after IR and localized in the cell nucleus. Furthermore, the results of GEPIA online data analysis were also certified that there was a correlation between HNF1A and H2AFX (P = 0.043) (Fig. 5B). In TCGA and GTEx databases, H2AFX expression in ESCC tumor tissues was higher than that in normal tissues, showing statistical significance (Fig. 5B). Next, we observed the effect of ectopic expression of HNF1A on γH2AX. We found no significant difference in γH2AX expression before IR between HNF1A group and NC group by western blot and immunofluorescence techniques (red fluorescence represents γH2AX) (Fig. 5C, D). However, after IR, we could clearly observe a significantly increase in γH2AX protein expression and γH2AX focus formation in both groups, especially in HNF1A group (Fig. 5C, D).

We explored the interactive mechanism of HNF1A on γH2AX, and found that HNF1A may play a role through the activation of PI3K/AKT signaling pathway. The results showed that the expression of PI3K p100β/p-AKT proteins increased after overexpression of HNF1A with or without IR (Fig. 5C). These data suggested that radiation and overexpression of HNF1A activated the PI3K/AKT signaling pathway, leading to cellular radiation-resistance. In addition, we hypothesized that HNF1A possibly increased γH2AX expression by actuating PI3K/AKT signaling pathway.

## Discussion

In China, more than 90% of esophageal cancer patients have ESCC 2. Although the overall prospects for patients with esophageal cancer have improved over the past 30 years, the majority of patients still have terminal cancer and poor prognoses. Patients receiving chemoradiotherapy with or without surgery survived for 2 years and had no recurrence less than 50% 21. The main causes of treatment failure after radiotherapy were local recurrence and distant metastasis. Therefore, how to improve esophageal cancer radiosensitivity became the focus.

The recent study showed that patients with pancreatic ductal adenocarcinoma (PDAC) with the HNF1A-positive subtype had the best prognostic survival time 22. In a separate study, patients of PDAC in high HNF1A group demonstrated an improvement in OS and DFS compared with the moderate/low HNF1A groups 9. In contrast, the patients of ESCC with high expression of HNF1A had a poor OS and PFS through online analysing by GEPIA and Kaplan-Meier plotter. The opposite phenomenon may be due to different tumor types, but a large amount of clinical data were needed to support our acquired datum. We also found that ESCC cells had more HNF1A expression than HEEC cells. It's worth noting that the combination of three liver transcriptional factors (TF), HNF1A, HNF4A, and FOXA3 transduced the Hepatocellular carcinoma (HCC) cell lines to more stably suppress cell proliferation 12. Interestingly, a study found that HNF1A was highly expressed in pancreatic cancer (PC) tissues, and that overexpression of HNF1A promoted PC cells proliferation and inhibited its apoptosis 23. These debates made us more interested in the biological function of HNF1A in ESCC cells. Our results showed that the overexpression of HNF1A promoted the proliferation of ESCC cells. In several studies, HNF1A had been linked to resistance to chemical drugs, including colon cancer 24 and pancreatic cancer 8-10. However, there was little research on its relationship with radiosensitivity. Thus, our study found that ectopic expression of HNF1A reduced radiosensitivity in ESCC cells and the radiobiological parameters SER in HNF1A group was decreasing.

HNF1A had been reported to be associated with tumor cell invasion, and inhibition of HNF1A reduced the invasion ability of cervical cancer cells 11, while increasing HNF1A improves liver metastasis of colorectal cancer 25. HNF1A was demonstrated to promote the invasion of ESCC cells in our results by transwell assay with or without IR. MMP2/MMP9 was recognized as an index that represents the invasion of cancer cells 26, 27. In various tumors, increased expression of MMP family proteins promoted the invasion of cells 28-30. EMT was a developmental process whereby stationary, adherent cells acquire the ability to migrate, and could drive metastasis, tumor recurrence, and therapy resistance in the context of cancer 31. Recently, silencing miR-101 promoted cell migration, while overexpression of miR-101 inhibited EMT and cell migration in ovarian cancer cell lines through the regulation of ZEB1 32. Our results showed that ectopic expression of HNF1A promoted the expression of Vimentin/N-cadherin/MMP2/MMP9 proteins, and inhibited the expression of E-cadherin proteins, thereby activating the EMT process to increase cell invasion.

Recently, study had found that pancreatic cancer cells were treated with doxycycline for two days, and PCR arrays were used to detect cell cycle and apoptosis-related genes. After HNF1A overexpression, 51 cell cycle genes and 11 apoptotic genes were significantly downregulated 8. Notably, in another study, HNF1A was highly expressed in pancreatic cancer tissues and inhibited the apoptosis of pancreatic cancer cells 23. The role of HNF1A in cancer is controversial, based on anterior research. However, our results supported that overexpression of HNF1A increased the radiation-induced apoptosis resistance of ESCC cells. To further verify our findings, BAX/BCL2 proteins were detected using western blot. The results showed that after IR, BAX proteins expression was decreased in HNF1A group compared with NC group, meanwhile BCL2 proteins was inverse. BAX/BCL2 proteins were ideal markers for the detection of apoptosis. A large number of studies had shown that BAX promoted the occurrence of apoptosis and that BCL2 inhibited the occurrence of apoptosis 33, 34. Furthermore, TE1 and KYSE150 cell cycles were blocked in the G0/G1 phase after IR, but this blockade was reduced after the overexpression of HNF1A. It was noted that the G2/M phase of HNF1A group did not change significantly after IR. Recently, a study showed that radiation-induced increased expression of FBXO31 was followed by selective degradation of Cyclin D1 leading to G0/G1 phase arrest 35. Consistent with their results, Cyclin D1 and CDK4 proteins expression decreased after IR inTE1 and KYSE150 cells, but ectopic HNF1A expression reversed this phenomenon. Moreover, CDK4-CDK6 inhibitors arrested cells in the G1/G0 phase, and Cyclins (D1, D2 and D3) assemble with CDK4 or CDK6 subunits in combinatorial and tissue-specific ways, thereby coordinating the inhibition of the activity of many genes needed to enter the S phase and subsequent cell cycle processes 36, 37. These date further demonstrated the reliability of our results.

The main biological effect of radiotherapy was the rapidly killing of proliferating cancer cells by inducing DNA damage beyond the ability of cell repair. In most cases, DNA DSBs are considered to be the most harmful form of DNA damage 38. The γH2AX protein was one of the most widely used biomarkers of DNA damage 39, 40 and participated in the process of DNA damaged repair 41-43. According to the published literature, no studies had shown a relationship between HNF1A and γH2AX, or even with DNA damage. Our experimental results preliminarily showed that HNF1A and γH2AX had the same trend with IR time and dose in TE1 and KYSE150 cells, and the expression of γH2AX was enhanced in HNF1A group. Class Ⅰa PI3K included the regulatory subunit p85 (α or β) and the catalytic subunit p110 (α, β, γ or δ), and the activity of p110 was regulated by p85 to activate the PI3K/AKT signaling pathway 44. Multiple studies had shown that radiation activated the PI3K/AKT/mTOR signaling pathway in a variety of tumor cell types, including human glioblastoma, cervical cancer, colorectal cancer and nasopharyngeal carcinoma 45-48.Our results were similar in that expression of PI3K p110β/p-AKT proteins were increased after IR. Actually, the activated PI3K/AKT signaling pathway inhibited apoptosis to promote cell survival and growth, leading to further radiation resistance 49. In our results, after ectopically expressing HNF1A, ESCC cells activated the PI3K/AKT signaling pathway to promote cell proliferation and invasion, and eventually lead to radiation resistance by preventing radiation-induced apoptosis and initiating DNA damage repair mechanisms. Although the PI3K/AKT signaling pathway was activated in the NC group after IR, the cellular activity was ultimately inhibited. After IR, abnormal activation of PI3K/AKT signaling pathway might be related to radiation-resistance. Interestingly, in pancreatic cancer cells, overexpression of HNF1A activated PTEN protein, thereby preventing AKT/mTOR phosphorylation and cell proliferation 10. This was still reasonable because identical kind of protein had different biological functions in different types of cancer.

## Conclusion

This study preliminarily demonstrated that HNF1A promoted the proliferation and invasion of ESCC cells, and weakened the radiosensitivity of cells by reducing G0/G1 phase arrest and decreasing apoptosis. In addition, the possible mechanism by which HNF1A played a role in radiation-resistance was through activation of the PI3K/ AKT signaling pathway and initiation of DNA damage repair. Therefore, our findings provided new insights into the role of HNF1A in ESCC and shed light on the regulation of HNF1A in the radiosensitivity and progression of ESCC. It provided theoretical basis for HNF1A to be a potential target for the treatment of ESCC.

## Abbreviations

HNF1A: Hepatocyte nuclear factor 1-alpha
IR: irradiation
ESCC: esophageal squamous cell carcinoma
NC: negative control
OS: overall survival
PFS: progression-free survival
SER: sensitization enhancement ratio
PC: pancreatic cancer
PCSC: pancreatic cancer stem cells
PDA: pancreatic ductal adenocarcinoma
DSBs: double-strand breaks
